# Characterization of the complete plastid genome of *Lysimachia christinae* Hance (Primulaceae)

**DOI:** 10.1080/23802359.2020.1863873

**Published:** 2021-01-27

**Authors:** Cai-Yun Zhang, Hai-Fei Yan, Feng-Ying Wang

**Affiliations:** a Guangdong Food and Drug Vocational College, Guangzhou, China; b Key Laboratory of Plant Resources Conservation and Sustainable Utilization, South China Botanical Garden, Chinese Academy of Sciences, Guangzhou, China; c Shanghai Chenshan Botanical Garden, Shanghai, China

**Keywords:** *Lysimachia*, Illumina sequencing, plastome, phylogenetic analyses

## Abstract

*Lysimachia christinae* Hance is widely distributed in subtropical China at the elevational range from 500–2300 m. The species is an important medicinal herb for treating jaundice, urinary disorders, and the liver. Here, we sequenced and characterized the whole plastid genome of *L. christinae*. It is 154,810 bp in length, containing two copies of inverted repeat (IR) regions (26,034 bp, each), a large single-copy (LSC) region (84,809 bp), and a small single-copy (SSC) region (17,933 bp). It has 114 genes, of which 80 are protein-coding, 30 are tRNA, and 4 are rRNA genes. The ML tree indicates *L. christinae* is closely related to *Lysimachia congestiflora* Hemsl. This genome information can help us better construct a backbone phylogeny of *Lysimachia* in the future.

*Lysimachia* L. is a large genus in the northern hemisphere in the family Primulaceae, containing 211 described species, with ∼81% occurring in East Asia (Yan et al. 2018). *Lysimachia christinae* Hance is widely distributed in the subtropical region in China growing at the elevational range from 500–2300 m (Hu and Kelso 1996). The species is a famous medicinal herb that is widely used to treat jaundice, urinary disorders, and the liver (Chen and Hu 1979). In this study, we characterized the complete plastid genome of *L. christinae* to determine its genome structure and evolutionary relationship to other Primulaceae species.

Fresh leaves of an individual of *L. christinae,* collected from Gulin County of Sichuan Province (28°16′01′′N, 105°49′39′′E), were used for DNA extraction with a modified CTAB protocol (Doyle and Doyle 1987). The voucher specimen (no. Y2011046) was deposited at the Herbarium of South China Botanical Garden. The Illumina paired-end (PE = 150 bp) library was constructed and sequenced on the Illumina Hiseq X Ten platform at the Beijing Genomics Institute (Wuhan, China). Low-quality reads and adaptor sequences were removed from ∼2 G bp raw data. The plastome of *L. christinae* was assembled using NOVOPlasty (Dierckxsens et al. 2017) with the reference plastome (NC_026197) of *Lysimachia coreana* Nakai (Son and Park 2016). The draft plastid genome was checked by remapping the clean reads in Geneious Prime 2019 (Biomatters, Ltd, Auckland, New Zealand). The tRNA genes were annotated using ARAGORN (Laslett and Canback 2004) and the coding genes with DOGMA (Wyman et al. 2004). Both genes were manually adjusted using Geneious Prime 2019. Other plastid genomes from the Primulaceae were downloaded from GenBank for the phylogenetic analyses. The aligned matrix comprised of 79 shared protein-coding genes extracted from 27 whole plastid genomes (including the newly generated plastome herein) was implemented in MAFFT (Katoh and Standley 2013), and used for ML tree construction. The maximum-likelihood (ML) tree was constructed using RAxML (Stamatakis 2014) with 1000 rapid bootstrap replicates.

The plastid genome of *L. christinae* (GenBank accession number MT982171) is 154,810 bp in length with a typical quadripartite structure. It contains two copies of inverted repeat (IR) regions (26,034 bp), a large single-copy (LSC) region (84,809 bp), and a small single-copy (SSC) region (17,933 bp). A total of 114 unique genes were encoded, of which 80 are protein-coding genes, 30 are tRNA genes, and four are rRNA genes. The overall GC content of the genome is 37%. Specifically, the GC values of the LSC, SSC, and IR regions are 34.8, 30.4, and 42.8%, respectively. The plastid genome structures of all three *Lysimachia* species are highly conserved since no genome rearrangement was detected (Son and Park 2016; Li et al. 2019). However, *Lysimachia coreana* (belonging to the subgen. *Palladia*) has only 79 protein-coding genes in its plastome, while both from the subgen. *Lysimachia* – *Lysimachia congestiflora* Hemsl. and *L. christinae* – contain 80 protein-coding genes (Li et al. 2019). The ML tree showed the species of *Lysimachia* formed a monophyletic clade with 100% bootstrap support value (Figure 1). *Lysimachia congestiflora* and *L. christinae* were recovered in a sister relationship with full support (bootstrap value = 100%), both belonging to the subgen. *Lysimachia* (Yan et al. 2018). The robust sister relationship between the subgen. *Lysimachia* and the subgen. *Palladia* was confirmed in this study, in agreement with the results of Anderberg et al. (2007) and Yan et al. (2018).

**Figure 1.**
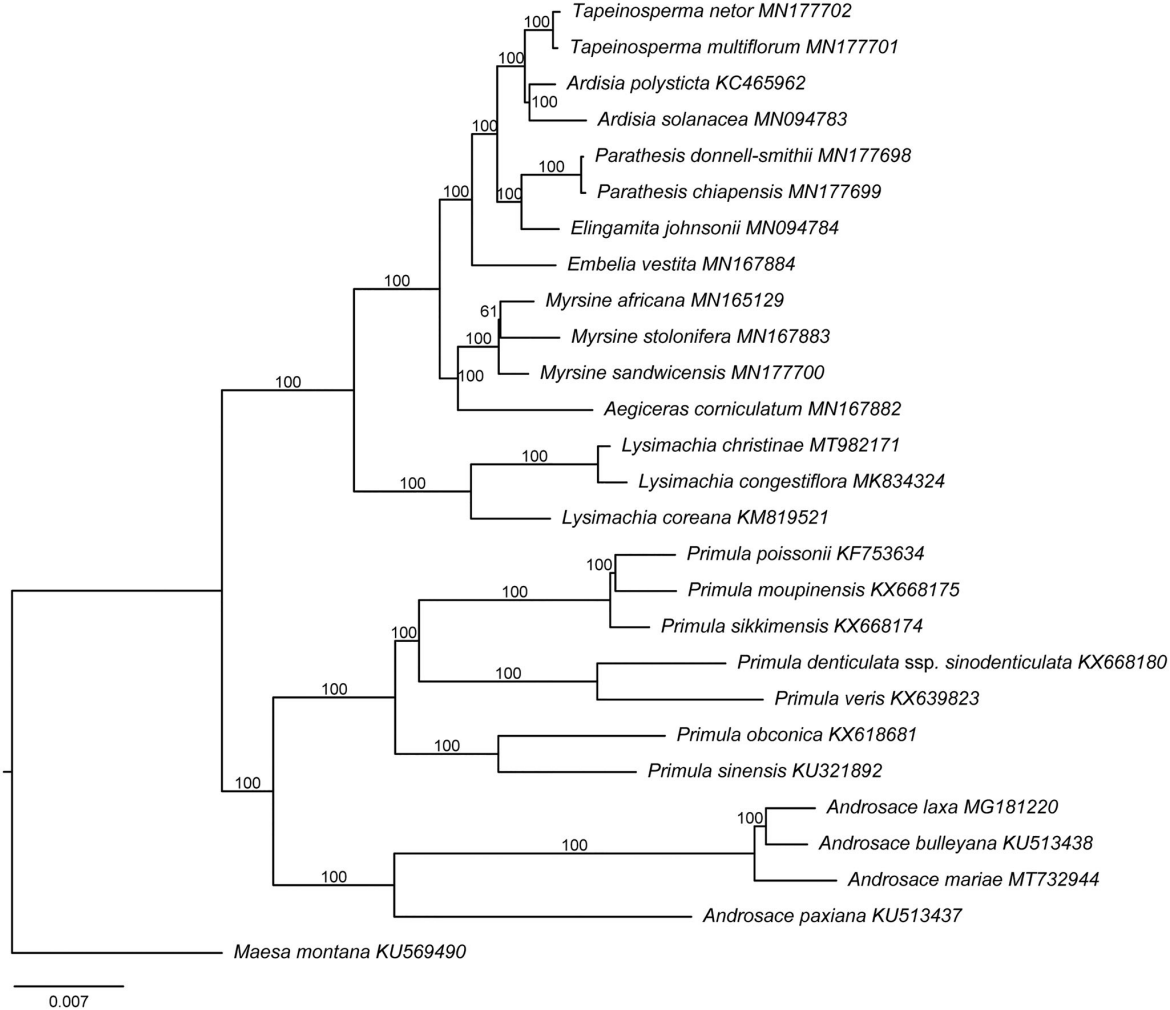
Phylogenetic relationships of Primulaceae inferred from maximum likelihood method based on 79 shared protein-coding genes. The node labels are the ML bootstrap values based on 1000 replicates.

## Data Availability

The data that support the findings of this study are openly available in GenBank at https://www.ncbi.nlm.nih.gov/, reference number MT982171. The raw sequencing data is available under GenBank Bioproject PRJNA670596.
